# Quality assessment of anterior composite restorations: clinical examination versus three digital photographic techniques

**DOI:** 10.1007/s00784-026-06870-3

**Published:** 2026-04-18

**Authors:** Aleyna Yazicioglu, Gul Yildiz Telatar

**Affiliations:** 1 Department of Restorative Dentistry, Trabzon Oral and Dental Health Center, Trabzon, Turkey; 2 Department of Restorative Dentistry, Faculty of Dentistry, Burdur Mehmet Akif Ersoy University, Burdur, Turkey

**Keywords:** Anterior composite restorations, Digital photography, FDI criteria, Lens-equipped smartphone, Teledentistry

## Abstract

**Objective:**

The aim of this study was for the first time to evaluate of intraoral digital photography in assessing buccal surface of anterior composite restorations using a smartphone (iPhone 14 Pro), a smartphone with a lens (2IN1 Phone Macro Lens), and a digital camera with a macro lens (Canon Rebel XTi), compared to clinical examination, based on World Dental Federation (FDI) criteria.

**Methods:**

A total of 185 anterior composite restorations were evaluated by calibrated restorative dentistry specialists. Restorations were scored according to the FDI criteria as intact, requiring repair, or needing replacement. Clinical examination was considered the gold standard. Photographs were taken under standardized conditions, and inter-observer and inter-method agreement were analyzed using Cohen’s Kappa and intraclass correlation coefficient (ICC).

**Results:**

High inter-observer agreement was observed across all methods (Kappa = 0.928–1.0). Good to excellent agreement was found between clinical examination and digital photography methods for FDI final scores (Kappa = 0.775–0.973, *p* < 0.001). Photographs taken with the lens-equipped smartphone and macro camera showed higher agreement with clinical examination (Kappa = 0.973).

**Conclusion:**

Digital photography, particularly with a lens-equipped smartphone and macro camera, offers effectiveness comparable to clinical examination in evaluating anterior composite restorations.

**Clinical relevance:**

This study demonstrates that intraoral digital photography, particularly with a lens-equipped smartphone and macro camera, achieves diagnostic outcomes closely aligned with clinical examination using FDI criteria. While smartphones alone are practical, lens-assisted imaging enhances accuracy, supporting its use as a cost-effective alternative to professional macro cameras.

## Introduction

The clinical success of anterior composite restorations remains a major topic of interest in restorative dentistry due to their functional and esthetic demands, as well as their susceptibility to failure over time [1]. With the increasing emphasis on minimally invasive and evidence-based dentistry, a growing number of clinical studies have focused on the longevity, failure modes, and evaluation methods of composite restorations, particularly in the esthetic zone [2].

Despite significant advances in adhesive systems and composite resin technology, composite restorations still present a limited clinical lifespan [2, 3]. In anterior teeth, the primary reasons for restoration failure differ from posterior regions and are not limited to secondary caries. The most frequent cause of restoration failure is fracture of the tooth or restorative material, whereas esthetic-related failures such as color changes, loss of anatomical form, and surface staining are more commonly associated with restorations placed for esthetic indications [1].

When defects such as marginal deterioration, minor fractures, or surface discoloration are present without underlying secondary caries, repair of the existing restoration may be a conservative and clinically acceptable option [4]. However, the decision between monitoring, repair, or replacement often depends on the clinician’s subjective judgment and clinical experience, which may lead to variability in treatment decisions [5].

Standardized clinical criteria, such as those developed by the World Dental Federation (FDI) and the modified United States Public Health Service (USPHS)/Ryge system, are commonly used to assess restoration quality in both clinical practice and research [5, 6]. Although these criteria provide structured evaluation frameworks, their application in daily practice may be time-consuming and subject to inter-examiner variability, even among experienced clinicians [7, 8].

Digital photography has emerged as an alternative that reduces bias in evaluating restoration quality. One of its primary uses is to allow clinicians to photograph restorations and send them to impartial researchers for assessment [9].

In the context of teledentistry, intraoral photography is recognized as a reliable diagnostic method for evaluating dental caries, trauma, restorations, periodontal diseases, oral lesions, and orthodontic conditions [9, 10]. In addition, this approach improves access to oral healthcare, reduces unnecessary clinical visits, and facilitates communication between clinicians and specialists [11, 12]. Digital photographic records also support patient education and provide standardized documentation for clinical and medico-legal purposes [13].

Modern technology has found its place in the medical world, as in every aspect of our lives. These advancements have created a new field called teledentistry, where telecommunications and dentistry are used together [14]. Dental photography requires specific technical precision due to the challenging conditions of the intraoral environment. Today, to achieve ideal image quality, interchangeable-lens cameras equipped with macro lenses offering 1:1 magnification are used in combination with light sources such as macro ring flashes. However, these systems are both costly and bulky, presenting disadvantages [15].

In contrast, the widespread use of smartphones offers a low-cost and practical photography solution, although image quality may vary between devices [16]. Smartphone megapixels are not equivalent to those of Digital Single-Lens Reflex (DSLR) cameras because smartphones have smaller camera sensors [17]. However, the cost of a smartphone with a detachable telephoto lens setup is approximately half that of a DSLR. A telephoto lens enables zooming without reducing image resolution and increases focal length instead of cropping and enlarging the image. All iPhone models starting from the iPhone 11 Pro are equipped with a telephoto lens [18].

This study is the first to investigate the validity of intraoral digital photography in evaluating anterior composite restorations. Our hypothesis is that photographs taken with a smartphone, a lens-equipped smartphone, and a macro-lens camera will yield results comparable to clinical examination.

The null hypothesis was that there would be no significant difference between clinical examination and photographic assessment methods in the evaluation of anterior composite restorations.

## Materials and methods

### Study design

This study is a validation study examining the use of intraoral digital photography in evaluating anterior composite restorations. The study was deemed scientifically and ethically appropriate by the Non-Interventional Clinical Research Ethics Committee of Recep Tayyip Erdogan University Faculty of Medicine under decision number 2023/137. Participants signed an informed consent form explaining the study’s purpose, content, and methods. The study was registered at ClinicalTrials.gov (Identifier: NCT07405619).

### Participants and sample

The study included 185 anterior teeth with composite restorations from individuals aged 18–64 who applied to the Department of Restorative Dentistry at XXX University. Restorations located on cervical, proximal, or incisal surfaces were eligible. Primary teeth, unrestored teeth, and teeth with amalgam, glass ionomer, or indirect restorations were excluded.

### Evaluation methods

Restorations were classified according to FDI criteria as intact (scores 1–3), requiring repair (score 4), or needing replacement (score 5) [5]. Clinical examination was considered the gold standard.

Photographs were taken using an iPhone 14 Pro, an iPhone 14 Pro equipped with a 2IN1 Phone Macro Lens, and a Canon Rebel XTi camera with a 100 mm macro lens. Detailed specifications, manufacturers, and imaging parameters of the photographic systems are summarized in Table 1.

**Table 1:** Photographic equipment and standardized imaging parameters used in the study

Device	Manufacturer	Camera	Lens	Lighting system	Distance	Key settings
iPhone 14 Pro	Apple Inc. (USA)	48 MP main, 12 MP ultra-wide	Built-in	Standardized LED (28 W matte white)	10 cm	Autofocus (tap-to-focus), HDR off, digital zoom off
iPhone 14 Pro + Macro Lens	Apple Inc. (USA); Guangdong Fengshang Intelligent Optoelectronics Co., Ltd. (China)	Same as above	2IN1 Phone Macro Lens (15×)	Integrated LED (53 LEDs; cold/warm/mixed; low/high)	10 cm	Autofocus, external illumination control
Canon Rebel XTi	Canon Inc. (Japan)	CMOS (3888 × 2592 px)	EF 100 mm f/2.8 Macro IS USM	Macro Ring Lite MR-14EX II (manual)	20 cm	Aperture f/22, ISO 200, shutter speed 1/160 s, white balance (flash), flash power 1/8

*LED* light-emitting diode, *ISO* ınternational organization for standardization, *MP* megapixel, *px* pixels

For each anterior restoration, one standardized buccal photograph was captured. A 360° photographic protocol was not performed, as the study aimed to evaluate diagnostic performance based on frontal images typically used in teledentistry screening.

For the iPhone 14 Pro, focus was achieved by tapping the screen to enable autofocus. Digital zoom and HDR mode were disabled.

Photographs were captured under standardized conditions, including the use of lip–cheek retractors and positioning the camera perpendicular to the tooth surface. Image acquisition was performed under standardized lighting using a 28 W matte white LED source. Ambient dental unit lighting was turned off to minimize reflections and variability. The working distance was maintained at approximately 10 cm for smartphone-based imaging and 20 cm for the macro camera system. Teeth were gently air-dried prior to imaging to improve surface visualization.

**Fig. 1:**
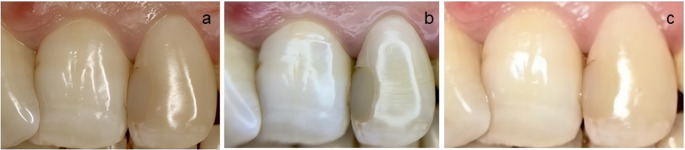
Images of the same restoration taken with different devices. a; image taken using a macro-lens camera., b; image taken using a lens-equipped smartphone, c; image taken using a smartphone

All photographs were saved in JPEG format and transferred to a computer for evaluation. The images were viewed using Windows Photo Viewer (Microsoft Corporation, Redmond, WA, USA). No digital manipulation, enhancement, or adjustment (e.g., brightness, contrast, color balance, or sharpness) was performed prior to assessment (Fig. 1).

### Calibration and evaluation

Evaluations were conducted by two restorative dentistry specialists. Calibration was performed on 20 anterior teeth with restorations, which were not included in the study, using both clinical and digital methods.

Clinical examination was performed using a dental mirror, a rounded-tip probe, and reflector light. Radiographic images (periapical or panoramic radiographs, when available) were reviewed only as adjunctive tools to exclude obvious secondary caries or periapical pathology. During the clinical examination, a dental mirror was used to allow comprehensive inspection of tooth surfaces. However, for the photographic protocol, only standardized buccal images of the anterior teeth were obtained and used for evaluation.

Standardized intraoral photographs were taken by a third experienced clinician, who was not involved in either the clinical or digital evaluations. The captured photographs were anonymized before assessment and were independently scored by two different restorative dentistry specialists according to the FDI criteria. The examiners evaluating the photographs were blinded to the clinical findings. Based on the FDI criteria, restorations were scored as follows: no intervention required (scores 1, 2, 3) with a final score of 1, repair required (score 4) with a final score of 2, and replacement required (score 5) with a final score of 3.

### Statistical analysis

The sample size was calculated using G*Power software (version 3.1.9.2; Heinrich Heine University, Düsseldorf, Germany). The calculation was based on effect size of 0.21, which was derived from previously published data [9]. With a significance level of α = 0.05 and a theoretical power of 0.80, the minimum required sample size was calculated as 175 restorations.

Inter-observer and inter-method agreement were assessed using Cohen’s Kappa statistic and the Intraclass Correlation Coefficient (ICC). Kappa was used for categorical variables, and ICC for quantitative variables, with a significance level of *p* < 0.05. Analyses were performed using IBM SPSS V23.

## Results

The mean age of the patients was 40.4 ± 1.3 years, with 50 female and 45 male participants. A total of 145 restorations (78.4%) were located in the maxilla and 40 restorations (21.6%) in the mandible. Regarding surface location, 44 restorations (23.8%) were cervical, 90 (48.6%) proximal, and 51 (27.6%) incisal.

### Interobserver agreement

The interobserver agreement for FDI final scores was found to be high across all imaging methods (Kappa = 0.928–1.0, *p* < 0.001).

Interobserver agreement was evaluated for each criterion according to the imaging methods (Table 2). Within the smartphone method, a statistically significant good level of agreement was observed between the observers for the criteria of material fracture, proximal contact point, surface gloss and texture, marginal discoloration, and color match. A statistically significant moderate level of agreement was found for marginal adaptation and anatomical form and contour. In both the smartphone with lens method and the macro photography method, a statistically significant good level of agreement was obtained between the observers for all evaluated criteria.

**Table 2:** Interobserver agreement by individual evaluation criteria

	Criterion	ICC (95% CI)	*p*
Smartphone	Material Fracture	0.796 (0.737–0.843)	< 0.001
	Marginal Adaptation	0.647 (0.554–0.723)	< 0.001
	Proximal Contact Point	0.76 (0.692–0.815)	< 0.001
	Form and Contour	0.665 (0.577–0.739)	< 0.001
	Surface Luster and Texture	0.763 (0.695–0.817)	< 0.001
	Marginal Discoloration	0.886 (0.85–0.913)	< 0.001
	Color Match	0.824 (0.771–0.865)	< 0.001
Smartphone + Lens	Material Fracture	0.85 (0.804–0.885)	< 0.001
	Marginal Adaptation	0.777 (0.713–0.829)	< 0.001
	Proximal Contact Point	0.82 (0.767–0.863)	< 0.001
	Form and Contour	0.82 (0.767–0.862)	< 0.001
	Surface Luster and Texture	0.813 (0.757–0.856)	< 0.001
	Marginal Discoloration	0.882 (0.845–0.91)	< 0.001
	Color Match	0.835 (0.786–0.874)	< 0.001
Macro Photography	Material Fracture	0.859 (0.815–0.892)	< 0.001
	Marginal Adaptation	0.76 (0.691–0.815)	< 0.001
	Proximal Contact Point	0.827 (0.775–0.867)	< 0.001
	Form and Contour	0.78 (0.716–0.83)	< 0.001
	Surface Luster and Texture	0.838 (0.79–0.876)	< 0.001
	Marginal Discoloration	0.895 (0.863–0.921)	< 0.001
	Color Match	0.852 (0.807–0.887)	< 0.001

### Comparison with clinical examination

Inter-observer agreement was determined to be at a good level. The methods were compared with clinical examination using Observer 2 as the reference.

Agreement between the methods was evaluated based on the final FDI scores. A good level of agreement was found between clinical examination and smartphone photography (Kappa = 0.775, *p* < 0.001), while very good agreement was observed with the smartphone plus lens and macro photography methods (Kappa = 0.973, *p* < 0.001) (Table 3).

**Table 3:** Comparison of clinical examination with other methods based on final FDI scores

Clinical						
					Kappa	*p*
		Intact	Repair	Replacement		
Smartphone	Intact	41 (91.1)	7 (17.1)	2 (2)	0.775	< 0.001
	Repair	4 (8.9)	26 (63.4)	4 (4)		
	Replacement	0 (0)	8 (19.5)	93 (93.9)		
Smartphone + Lens	Intact	45 (100)	0 (0)	0 (0)	0.973	< 0.001
	Repair	0 (0)	40 (97.6)	2 (2)		
	Replacement	0 (0)	1 (2.4)	97 (98)		
Macro Photography	Intact	45 (100)	0 (0)	0 (0)	0.973	< 0.001
	Repair	0 (0)	40 (97.6)	2 (2)		
	Replacement	0 (0)	1 (2.4)	97 (98)		

The agreement between clinical examination and other methods in terms of individual FDI criteria was evaluated (Table 4). A good level of agreement was found between clinical examination and the smartphone method for material fracture; moderate agreement was observed for marginal adaptation, proximal contact point, form and contour, caries at the restoration margin, surface gloss, surface texture, marginal staining, and color match; and low agreement was detected for hard tissue defects at the restoration margin. Between clinical examination and both the smartphone with lens and macro photography methods, good agreement was found for material fracture, caries at the restoration margin, and marginal staining; moderate agreement was observed for other criteria; and low agreement was found for hard tissue defects.

**Table 4:** Agreement between clinical examination and other methods based on FDI criteria (ICC with 95% Confidence Intervals)

	Criterion	ICC (95% CI)	*p*
Clinical- Smartphone	Material Fracture	0.822 (0.769–0.863)	< 0.001
	Marginal Adaptation	0.5 (0.383–0.6)	< 0.001
	Proximal Contact Point	0.591 (0.488–0.677)	< 0.001
	Form and Contour	0.519 (0.406–0.617)	< 0.001
	Caries at Restoration Margin	0.692 (0.608–0.76)	< 0.001
	Hard Tissue Defect at Restoration Margin	0.28 (0.141–0.407)	< 0.001
	Surface Luster and Texture	0.598 (0.497–0.683)	< 0.001
	Marginal Discoloration	0.737 (0.663–0.796)	< 0.001
	Color Match	0.633 (0.538–0.712)	< 0.001
Clinical- Smartphone + Lens	Material Fracture	0.86 (0.817–0.893)	< 0.001
	Marginal Adaptation	0.51 (0.395–0.609)	< 0.001
	Proximal Contact Point	0.622 (0.525–0.703)	< 0.001
	Form and Contour	0.605 (0.505–0.689)	< 0.001
	Caries at Restoration Margin	0.824 (0.772–0.865)	< 0.001
	Hard Tissue Defect at Restoration Margin	0.28 (0.141–0.407)	< 0.001
	Surface Luster and Texture	0.69 (0.607–0.759)	< 0.001
	Marginal Discoloration	0.765 (0.697–0.818)	< 0.001
	Color Match	0.691 (0.607–0.759)	< 0.001
Clinical- Macro Photography	Material Fracture	0.923 (0.898–0.942)	< 0.001
	Marginal Adaptation	0.542 (0.431–0.636)	< 0.001
	Proximal Contact Point	0.559 (0.451–0.651)	< 0.001
	Form and Contour	0.597 (0.496–0.682)	< 0.001
	Caries at Restoration Margin	0.824 (0.772–0.865)	< 0.001
	Hard Tissue Defect at Restoration Margin	0.28 (0.141–0.407)	< 0.001
	Surface Luster and Texture	0.705 (0.624–0.771)	< 0.001
	Marginal Discoloration	0.772 (0.706–0.824)	< 0.001
	Color Match	0.693 (0.61–0.761)	< 0.001

*ICC* intraclass correlation coefficient

### Failure detection

The agreement between clinical examination and other methods was evaluated (Table 5). A binary scoring system was used to calculate the overall failure rate: 0 = no failure, 1 = failure (repair or replacement). For failure detection, the smartphone method demonstrated 93.57% sensitivity, 91.11% specificity, 97.04% positive predictive value (PPV), and 82% negative predictive value (NPV). Both the smartphone with lens and macro photography methods exhibited excellent diagnostic performance with 100% sensitivity, specificity, PPV, and NPV.

**Table 5:** Evaluation of agreement between clinical examination and other methods

		Smartphone		Smartphone+ Lens		Macro Photography	
		No Failure	Failure	No Failure	Failure	No Failure	Failure
Clinical	No Failure	41 (91.1)	4 (8.9)	45 (100)	0 (0)	45 (100)	0 (0)
	Failure	9 (6.4)	131 (93.6)	0 (0)	140 (100)	0 (0)	140 (100)
Kappa		0.816		1		1	
p		< 0.001		< 0.001		< 0.001	
Sensitivity		%93.57		%100		%100	
Specificity		%91.11		%100		%100	
PPV		%97.04		%100		%100	
NPV		%82		%100		%100	

## Discussion

This study aimed to evaluate the effectiveness of intraoral digital photography in assessing anterior composite restorations, compared to clinical examination, using FDI criteria. No similar study in the literature has evaluated restoration quality in the context of teledentistry using a smartphone (iPhone 14 Pro), a lens-equipped smartphone (2IN1 Phone Macro Lens), and a macro-lens digital camera (Canon Rebel XTi). Thus, this study provides an original contribution by comparing the effectiveness of these three methods.

The results indicate high inter-observer agreement across all methods (Kappa = 0.928–1.0). The smartphone method showed slightly lower agreement compared to the lens-equipped smartphone and macro camera, likely due to the smartphone camera’s lower resolution and detail-capturing capacity [16]. Kohara et al. [16] used a smartphone and macro camera for occlusal caries detection, reporting Kappa values ranging from 0.60 to 0.66. In contrast, this study achieved higher Kappa values (0.775–0.973), which may be attributed to the technological superiority of the devices used (e.g., the iPhone 14 Pro’s 48 MP camera) and standardized imaging conditions.

Signori et al. [9] reported low to moderate agreement (Kappa = 0.12–0.34) for anterior restorations evaluated with an intraoral camera. However, this study found good to excellent agreement for anterior restorations (Kappa = 0.775–0.973). This difference may stem from the superior resolution of the images used in this study (2268 × 4032–5472 × 3648 pixels) compared to the intraoral camera’s resolution (1024 × 768 pixels). Additionally, the accessibility of the anterior region likely enhanced image quality and diagnostic accuracy.

Lower agreement was observed between methods for marginal adaptation, proximal contact points, and form/contour criteria. This may be due to the two-dimensional nature of digital photography, which lacks the perceptual capacity provided by tactile examination [19, 20]. Valizadeh-Haghi et al. [20] reported that smartphone photographs were inadequate for detecting marginal gap. Clinical examination with a probe allows for more precise evaluation of marginal adaptation [20]. This suggests that digital methods may be limited in criteria requiring detailed assessment.

Aesthetic criteria, particularly color match, were identified as a leading cause of restoration failure [21]. However, the evaluation of these criteria can vary based on clinicians’ experience and subjective perceptions. Simplifying the FDI criteria into three final scores (intact, repair, replacement) in this study may have increased inter-observer and inter-method agreement. This finding supports the idea that less complex scoring systems can provide more consistent results in clinical evaluations [22].

The smartphone method was effective in detecting obvious failures, such as material fractures, but limited in detailed assessments like marginal adaptation and proximal contact points. In contrast, the lens-equipped smartphone and macro camera provided results closely comparable to clinical examination, emerging as strong alternatives for teledentistry applications [18, 23].

Teledentistry has been shown to improve access to care, particularly in underserved and rural populations, where it facilitates screening, referral, and early diagnosis [12]. Furthermore, teledentistry and mobile health (mHealth) applications have been shown to support oral health promotion, enhance patient engagement, and facilitate remote consultations within dental care workflows [24].

Clinical studies have reported moderate to good agreement between smartphone-based photographic assessment and clinical examination for caries detection and the evaluation of anterior composite restorations under standardized conditions [20, 25].

Recent investigations evaluating smartphone-based intraoral photography report that diagnostic performance improves significantly when optical enhancement systems, such as macro lenses or controlled illumination, are incorporated [26]. This supports the findings of the present study, where lens-assisted smartphone imaging demonstrated results closely comparable to clinical examination.

Notably, the lens-equipped smartphone, offering high-resolution images, may serve as a cost-effective alternative to the macro camera. A study in plastic surgery reported that a lens-equipped smartphone (iPhone 11 + Moment 58 mm telephoto lens) performed similarly to a macro-lens DSLR camera [18]. Similarly, this study observed that the lens-equipped smartphone yielded FDI scores nearly equivalent to those of the macro camera.

Technological advancements, particularly improvements in smartphone camera systems, suggest that this method will become more widespread and reliable in the future. Future studies focusing on posterior restorations and comparisons of different devices can further expand these findings.

The limitations of the present study should be considered when interpreting the findings. Similar to the observations reported by Valizadeh-Haghi et al. (2023), photographic assessment showed inherent limitations for criteria requiring tactile examination due to the inability to use an explorer or probing [20]. Although image magnification may enhance the detection of visually evident defects, photographic artifacts can also lead to misinterpretation, particularly in the assessment of fractures. Additionally, the two-dimensional nature of photographs and tooth orientation may limit the visualization of certain restoration surfaces. Despite evaluator calibration based on FDI criteria, the subjective nature of aesthetic assessment remains a source of inter-evaluator variability. Finally, only buccal surfaces of anterior teeth were evaluated in the photographic protocol. While this approach may limit the assessment of palatal/lingual and interproximal surfaces, it reflects real-world teledentistry screening conditions, where standardized frontal images are most feasible. Therefore, this study design enhances external validity for remote dental screening applications.

## Conclusion

This study confirms that lens-equipped smartphone and macro photography can achieve good agreement with clinical examination for specific parameters, particularly material fracture and caries at restoration margins; however, agreement across most other FDI criteria was predominantly moderate, with consistently low agreement for hard tissue defects, indicating that these methods should be considered complementary rather than equivalent to clinical examination. While the iPhone 14 Pro stands out for its practicality and accessibility, its image quality is slightly inferior to that of the lens-equipped smartphone and macro camera. The lens-equipped smartphone holds high potential as a cost-effective alternative to the macro camera in teledentistry applications.

In teledentistry settings, digital buccal anterior photography may facilitate remote triage and consultation, potentially reducing the need for unnecessary clinical visits.

The authors declared no potential conflicts of interest with respect to the research, authorship, and/or publication of this article.

## Data Availability

Datasets related to this article are available upon request to the corresponding author.
